# Excision of Invasive Squamous Cell Carcinoma Complicated by Osteomyelitis and Tobacco Use: A Case Report

**DOI:** 10.7759/cureus.54629

**Published:** 2024-02-21

**Authors:** Harriet Kaye Austin, Gloria M Gamboa, Carlene Waters-Hollingsworth

**Affiliations:** 1 Plastic and Reconstructive Surgery, University of Central Florida College of Medicine, Orlando, USA; 2 Plastic and Reconstructive Surgery, Orlando Veterans Affairs Medical Center, Orlando, USA

**Keywords:** soft tissue necrosis, pseudomonas aeruginosa (p. aeruginosa), delayed wound healing, integra, fascio-cutaneous flap, acute osteomyelitis, squamous cell carcinoma of the skin

## Abstract

Squamous cell carcinoma (SCC) is a common type of skin cancer that can be treated through surgical excision using Mohs micrographic surgery (MMS) which results in minimal scarring and low complications. Soft tissue defects as a result of MMS that are too large to be primarily closed can be repaired with secondary intention healing through the use of biologic prosthetics that promote dermal regeneration and tissue remodeling with high success rates. Other non-surgical treatment options include chemotherapy (topical or systemic), radiation, or immunotherapy for advanced skin cancers. In this case, our patient is a 76-year-old male with a history of tobacco use who presented with ulcerative SCC and developed a necrotic soft-tissue infection of *Pseudomonas* *aeruginosa* complicated by calvarial osteomyelitis six weeks following wide excision of scalp SCC and wound defect repair with application of Integra® Bilayer Wound Matrix (Integra LifeSciences, Princeton, New Jersey, United States) to promote re-vascularization and tissue regrowth. The patient is currently recovering well after the excision of the necrotic scalp lesion and second-stage reconstruction with right scalp fasciocutaneous flap and full-thickness skin graft with proper antibiotic administration. Complications were likely due to delayed wound healing from post-operative cigar use increasing his risk for infection and application of biologic prosthetics that potentially served as a nidus for bacterial adherence and biofilm production of *P.* *aeruginosa,* which led to osteomyelitis, an exceedingly rare complication for patients that undergo MMS.

## Introduction

The most common complication associated with Mohs micrographic surgery (MMS) is postoperative infection; however, risks are usually <1% with the most common pathogens being methicillin-sensitive *Staphylococcus aureus* (MSSA) and methicillin-resistant *S.* *aureus* (MRSA)[1-3]. *Pseudomonas aeruginosa* is a gram-negative opportunistic pathogen that frequently targets patients in an immunocompromised state exposed to healthcare settings. *P. aeruginosa* production of biofilm is one of its essential virulence factors which enhances its pathogenicity and persistence. Previous studies with consistent findings show that exposure to cigarette smoke promotes biofilm production in addition to enhanced defense against oxidative stress which renders neutrophil killing to be ineffective [4]. Cigarette smoking impairs proper and timely wound healing through multiple pathways including (i) compromised nutritional and oxygenated blood flow to tissues, (ii) reduced proliferation of erythrocytes, macrophages, and fibroblasts, and (iii) decreased bactericidal activity through dysfunction of neutrophils, as previously mentioned [5]. Nicotine is the main constituent in cigarettes that induces tissue hypoxia due to its vasoconstrictive effects and increased platelet aggregation. Inhalation of carbon monoxide decreases optimal oxygen binding to hemoglobin leading to reduced oxygen content in the blood, further worsening tissue hypoxia. Across all surgical specialties, current and former smokers have a higher incidence rate of both infectious and noninfectious postoperative healing complications compared to nonsmokers [6].

Acute osteomyelitis can occur through contiguous spread from surrounding tissues, hematogenous dissemination, or inoculation of bone. Contiguous spread and inoculation of bone typically occurs in the setting of surgery or trauma. Persistent, untreated, or non-healing soft tissue infection can continuously spread to underlying bone whereas direct inoculation can occur during surgery that involves bone exposure or manipulation. Although calvarial osteomyelitis is a rare complication post MMS, with only three known cases reported in the literature, it is important to be aware of it to ensure prompt management before the setting of irreversible sequelae of infection [7].

## Case presentation

**Figure 1:**
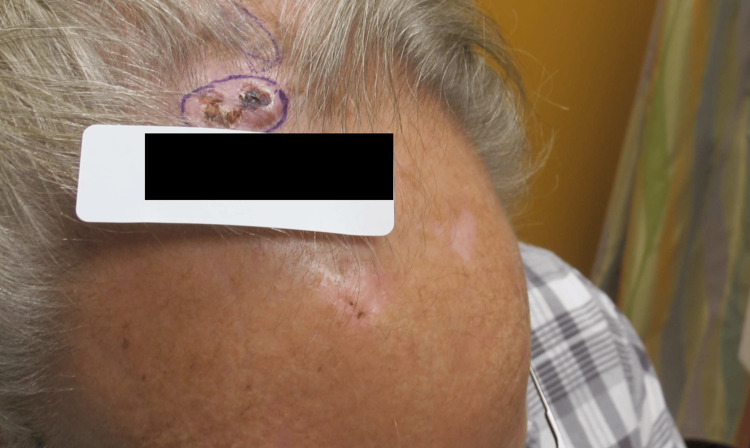
Ulcerative squamous cell carcinoma of scalp at initial consult

A 76-year-old male with a complex past medical history that was significant for tobacco use, abdominal aortic aneurysm s/p stent repair, coronary artery disease, and atrial fibrillation on apixaban presented to the clinic for evaluation and treatment for biopsy-proven squamous cell carcinoma (SCC) of the mid-frontal scalp (Figure 1). The patient stated that it was a new skin lesion that was biopsied three weeks prior to his initial consult, which showed findings of well-differentiated SCC. He reported pruritus and bleeding associated with the skin lesion. He denied unintentional weight loss, fevers, chills, headaches, visual changes, weakness, dizziness, chest pain, shortness of breath, nausea, vomiting, and abdominal pain. He denied a prior history of skin cancer lesions. He endorsed tobacco use of around seven cigars per week but denied alcohol, recreational drug use, vapor/inhalant use, and chewing tobacco. 

Upon physical exam, abnormal findings included a mid-scalp ulcerated skin lesion with irregular borders measuring 2.5 cm x 3.5 cm. After shared-decision making, he opted for surgical excision. His surgery date was scheduled four months after his initial consult with unremarkable preoperative labs and work-up results for wide excision of frontal scalp carcinoma with frozen section possible local flap, skin graft, or placement of Integra® Bilayer Wound Matrix (Integra LifeSciences, Princeton, New Jersey, United States). Repair of the defect after SCC excision was to be decided depending on the size of the defect after margins were confirmed to be negative. 

**Figure 2:**
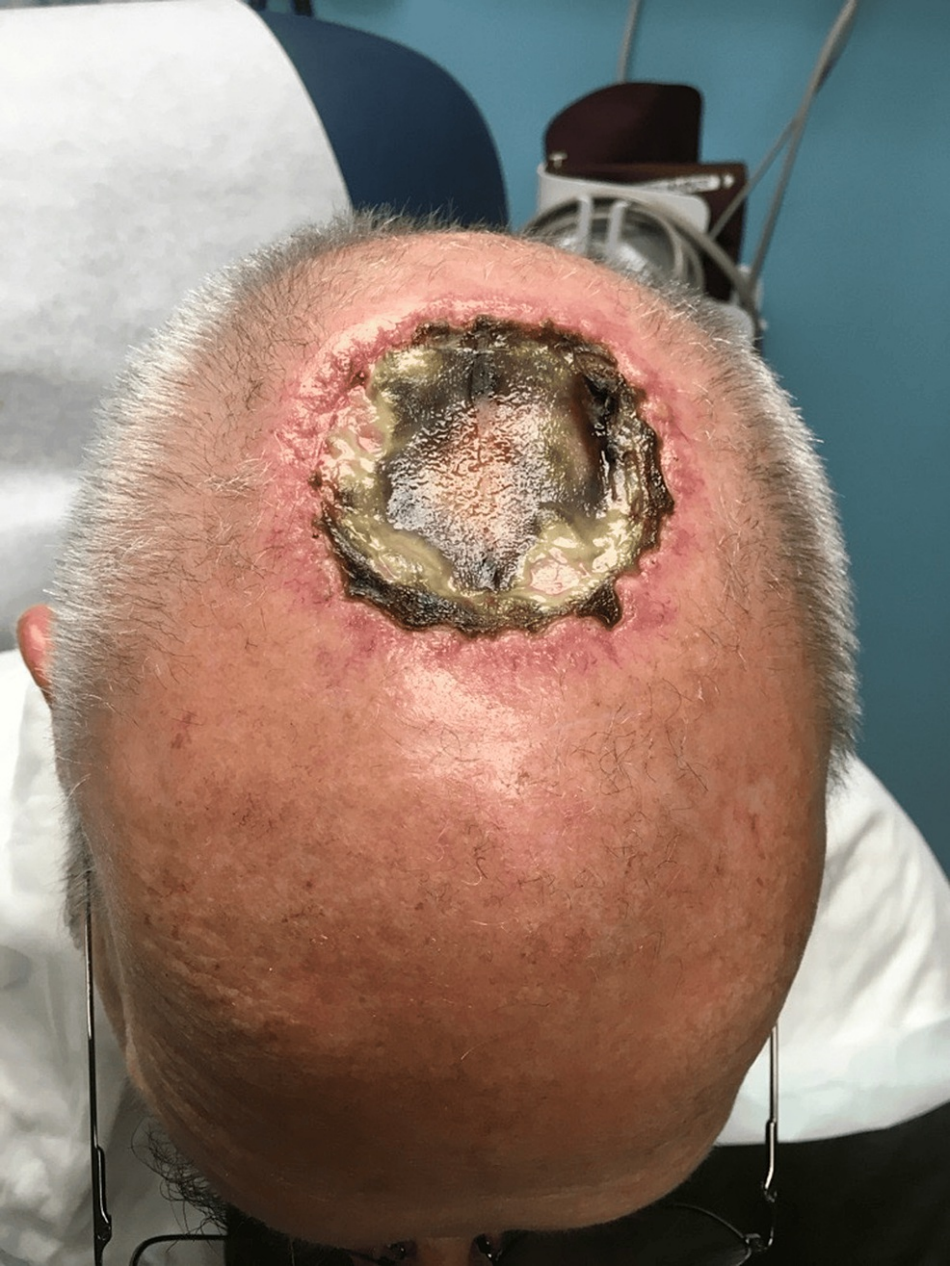
Week 3 postoperative vist after initial surgery with Integra®* placement *Integra® Bilayer Wound Matrix (Integra LifeSciences, Princeton, New Jersey, United States)

After excision, the initial defect measured 6.1 cm x 5.2 cm and the scalp wound was repaired with the use of fenestrated Integra®, which was tailored and fixated to the wound. Histopathological findings of the specimen showed invasive SCC with focal perineural invasion present. During his first two weekly postoperative visits, the patient was doing well with evidence of re-epithelialization and no complaints. However, he admitted to smoking cigars during his second postoperative visit. The patient was made aware of the adverse effects of tobacco on proper wound healing. Approximately three weeks following his initial surgery, his surgical wound showed significant black necrotic tissue with the presence of Integra® failure (Figure 2). He was afebrile, hemodynamically stable, and remained alert and oriented. His wife endorsed cleaning and placing new dressings daily. Other than the visualized black necrotic tissue he did not have any other signs or symptoms of infection including lack of rashes, fevers, chills, lymphadenopathy, nausea, vomiting, and abdominal pain.

**Figure 3:**
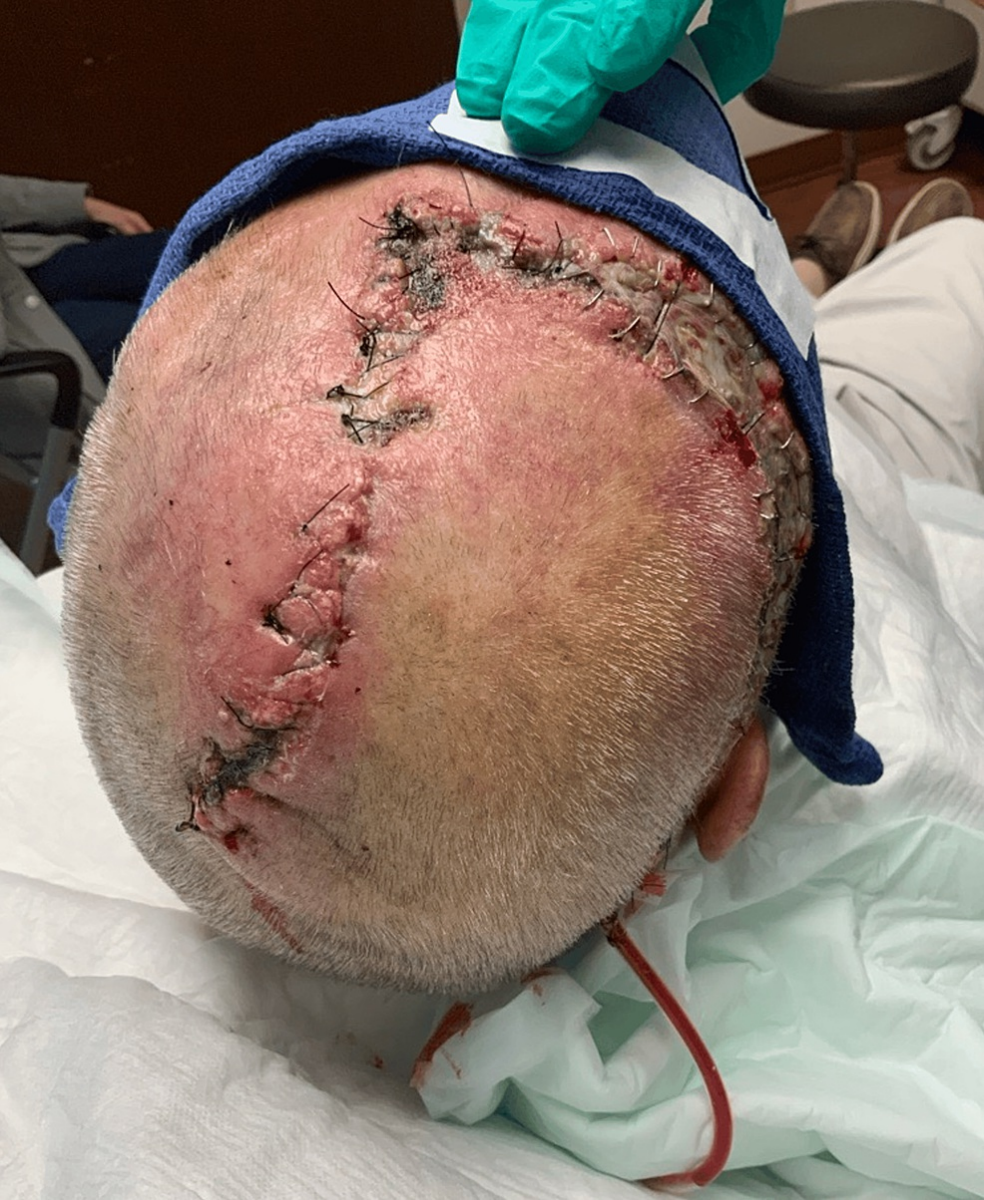
First postoperative follow-up after second stage reconstruction

**Figure 4:**
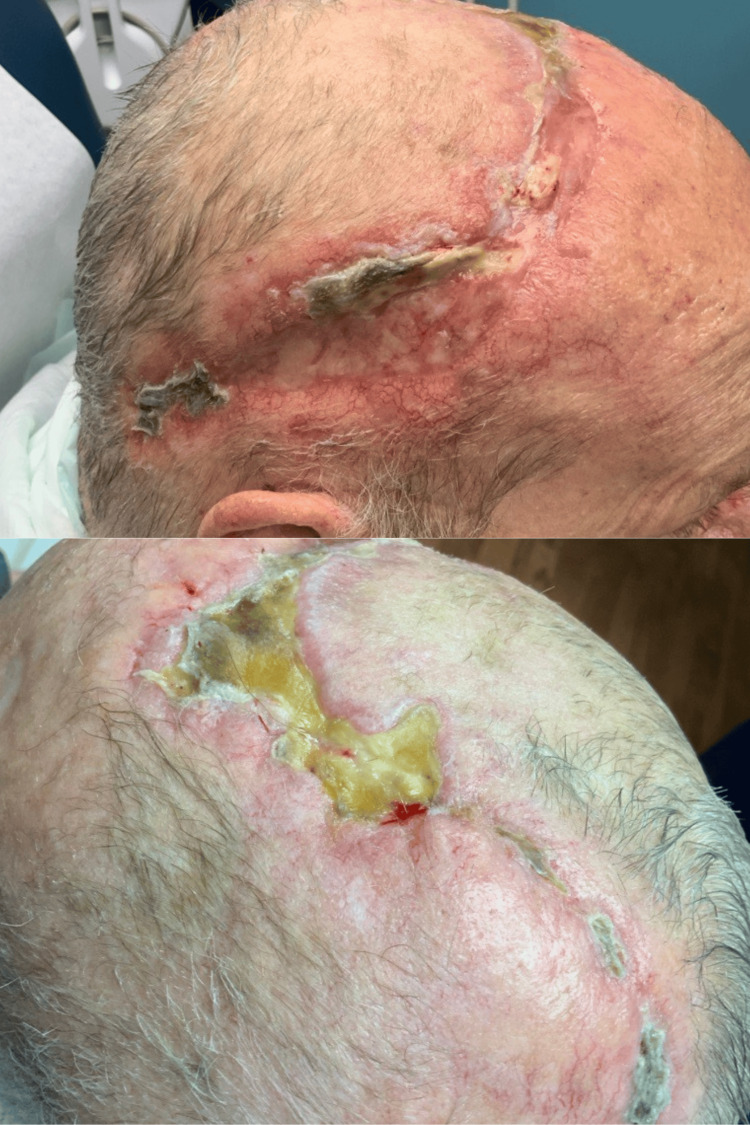
Multi-view of healing flap

He was started on oral antibiotics cephalexin twice daily for two weeks and was scheduled for second-stage scalp carcinoma excision and reconstruction with local flap and full-thickness skin graft. During his second procedure, the scalp was re-excised with a defect measuring 7 cm x 7 cm which was reconstructed with a flap and drain placement (Figure 3). The patient tolerated the procedure well and specimens of scalp tissue and a bone biopsy were sent for pathology and culture. Results of the specimen showed osteomyelitis of the skull and scalp tissue with necrosis and no residual SCC. The wound culture results taken intraoperatively were positive for the growth of *P.* *aeruginosa*. The patient completed two weeks of ciprofloxacin and was switched to IV cefepime for an additional four weeks to complete a total of six weeks of therapy for osteomyelitis and* Pseudomonas* wound infection. The patient continues to follow up with Infectious Disease and Plastic Surgery for the management of infection and wound care, respectively (Figure 4). 

## Discussion

MMS is a surgical procedure that usually has a low rate of adverse events and postoperative complications, but proper wound healing is an important factor in ensuring low complications and infection rates post MMS. Wound healing can be impeded by social behaviors including perioperative smoking. This patient presents with acute osteomyelitis likely secondary to a* P.* *aeruginosa* wound infection that contiguously spread into the adjacent skull. 

Although most post-MMS infections are associated with MSSA and MRSA, a study concluded that cigarette smoke promoted biofilm production of *P.* *aeruginosa* which may contribute to its virulence and persistence to become an invasive disease [4]. A different study on biologic prosthetics and biofilm production of *S.* *aureus* and *P.* *aeruginosa *showed that *P. aeruginosa* had stronger adhesion and biofilm formation compared to *S.* *aureus*, and these results were consistent with past findings [8]. The study stated that despite the decreasing bacterial viability of *P.* *aeruginosa *on the prosthetic surfaces, its continued biofilm production enhanced its persistence to remain on the biologic. However, the study was limited due to the limited type of biologics tested on human acellular dermis and porcine small intestinal submucosa, whereas the Integra® is made of bovine tendon collagen. It was summarized that the material of the biologic may impact the adhesion and growth of different types of bacteria.

In the case of the presented patient, his cigar-smoking behavior likely placed him in an immunocompromised state resulting in delayed wound healing and increased risk for infection. Moreover, the placement of Integra® may have assisted in propagating the invasion with *P.* *aeruginosa*. 

## Conclusions

Although MMS is a well-known surgical technique with a high cure rate and low complications, the potential for infection still exists. In this case, proper wound healing was delayed likely due to tobacco use which led to soft tissue infection complicated by acute osteomyelitis. There are several practical strategies to discourage high-risk behaviors such as smoking to allow for tissue regrowth and healing and prevent post-MMS rare complications such as calvarial osteomyelitis. It is highly recommended to include nicotine/cotinine tests in preoperative work-ups in higher-risk patients initiating the test closer to the scheduled surgery along with close monitoring and follow-ups postoperatively to track the progress of the healing trajectory of the wound. With emphasis on proper patient education about the adverse effects of tobacco use on wound healing, complications related to post-MMS infection can be further minimized. 
